# Surgery at a Discount

**Published:** 1848-08

**Authors:** 


*Surgery at a Discount.*—The following case came before one of the city magistrates a few days since. The son of a man whose income is say $25,000 a year, received a wound about three-fourths of an inch long on the upper lip. The wound extended obliquely upwards and backwards from the free border of the lip, which was entirely divided for the space of a quarter of an inch. A young physician brought the edges together by means of the twisted suture, and adhesion took place immediately. The reparation is almost perfect, scarcely a mark being left in the line of the wound. After the lapse of a proper time, a bill of *ten dollars* was sent to the boy's father, payment was refused; and suit was brought to enforce payment. One of the editors of this Journal swore (and does yet for that matter) that the service was well worth ten dollars; another physician swore that it was worth just three dollars; and a student of medicine (!) swore that it was worth just three dollars. The doctor's testimony being as 1½ to 1, the court decided that the cure of accidental hare-lip is worth just three dollars! The plaintiff, not satisfied with the judgment, took an appeal. He says he wants the value of such a piece of surgery fixed and settled undubitably; wants it placed on record, so that in future there may be no litigation. We shall give notice of the result.—*Louisville Western Med. Journal.*

